# Diversity of Retinal Ganglion Cells Identified by Transient *GFP* Transfection in Organotypic Tissue Culture of Adult Marmoset Monkey Retina

**DOI:** 10.1371/journal.pone.0054667

**Published:** 2013-01-15

**Authors:** Satoru Moritoh, Yusuke Komatsu, Tetsuo Yamamori, Amane Koizumi

**Affiliations:** 1 Department of Cell Physiology, National Institute for Physiological Sciences, Myodaiji, Okazaki, Japan; 2 Division of Brain Biology, National Institute for Basic Biology, Myodaiji, Okazaki, Japan; 3 Department of Physiological Sciences, School of Life Science, The Graduate University for Advanced Studies (SOKENDAI), Okazaki, Japan; 4 Department of Molecular Biomechanics, The Graduate University for Advanced Studies (SOKENDAI), Okazaki, Japan; National Tsing Hua University, Taiwan

## Abstract

The mammalian retina has more diversity of neurons than scientists had once believed in order to establish complicated vision processing. In the monkey retina, morphological diversity of retinal ganglion cells (RGCs) besides dominant midget and parasol cells has been suggested. However, characteristic subtypes of RGCs in other species such as bistratified direction-selective ganglion cells (DSGC) have not yet been identified. Increasing interest has been shown in the common marmoset (*Callithrix jacchus*) monkey as a “super-model” of neuroscientific research. Here, we established organotypic tissue culture of the adult marmoset monkey retina with particle-mediated gene transfer of *GFP* to survey the morphological diversity of RGCs. We successfully incubated adult marmoset monkey retinas for 2 to 4 days *ex vivo* for transient expression of *GFP*. We morphologically examined 121 RGCs out of more than 3240 *GFP*-transfected cells in 5 retinas. Among them, we identified monostratified or broadly stratified ganglion cells (midget, parasol, sparse, recursive, thorny, and broad thorny ganglion cells), and bistratified ganglion cells (recursive, large, and small bistratified ganglion cells [blue-ON/yellow-OFF-like]). By this survey, we also found a candidate for bistratified DSGC whose dendrites were well cofasciculated with ChAT-positive starburst dendrites, costratified with ON and OFF ChAT bands, and had honeycomb-shaped dendritic arbors morphologically similar to those in rabbits. Our genetic engineering method provides a new approach to future investigation for morphological and functional diversity of RGCs in the monkey retina.

## Introduction

The mammalian retina has more diversity of retinal neurons than scientists had once believed in order to establish complicated vision processing [1], [2]. In the rodent retina, recent genetic engineering approaches have revealed more diverse populations of retinal ganglion cells [3], [4], [5], [6]. Even in the monkey retina, Dacey and other researchers showed morphological diversity of retinal ganglion cells, although midget and parasol cells were dominant [7], [8]. Extensive survey of the diversity of retinal ganglion cells in the monkey retina has been limited because of genetic engineering limitations and limited resource availability. Increasing interest has been shown in the monkey retina as a non-human model animal for neuroscience, and the common marmoset (*Callithrix jacchus*) monkey has become a “super-model” of neuroscientific research [9] and also retinal neuroscience [10], [11], [12], [13], [14], [15], [16], [17], [18], [19], [20], [21], [22] because the marmoset is relatively inexpensive and has a high reproduction rate. Previously, intraocular-injection of an AAV-virus vector in the eyes of a marmoset has been conducted for gene transfection, but several months were needed for the expression of ectopic transgenes [16]. When incubation and transient gene expression methods *ex vivo* for the marmoset monkey retina have been established, the methods should be powerful genetic engineering tools *ex vivo* for investigating monkey retinas. The aim of this study was to establish an organotypic tissue culture of the adult marmoset monkey retina with a genetic engineering technique of transient gene expression for surveying morphological diversity of RGCs.

## Materials and Methods

### Animals

Experiments were carried out on four (one male and three females) adult common marmosets (*Callithrix jacchus*), ranging in weight from 250 to 300 g, obtained from CLEA Japan Inc. and maintained in the Marmoset Research Facility, National Institute for Basic Biology (NIBB) Bioresouce Center. Animals were housed indoors at Marmoset Research Facility of NIBB, in stainless lattice cages (69×65×72 cm or 104×65×154 cm) that permitted auditory and olfactory contact between animals in different groups. The cages contained fabric hammocks, wooden perches and nest boxes. The animals were fed CMS-1M (CLEA Japan, Inc., Tokyo, Japan) supplemented with vitamin D, vitamin C, Lactobacillus preparation and honey at 12∶30–13∶30 h; in addition, food was usually available in the cages at all times. Marmoset jelly (Mazuri, Richmond, IN), boiled quail eggs, Calorie Mate block (Otsuka Pharmaceutical Co., Ltd) and apples were provided as daily special additional diet. Water was available ad libitum. Lights were on from 6∶30 to 18∶30 h, and room temperature and humidity were maintained at approximately 24°C and 30–70%, respectively.

All procedures were carried out according to the provisions of the National Institute for Physiological Sciences (NIPS) code of practice for the care and use of animals and were approved by the institutional ethics committee of NIPS (No. 11A172) and by Animal Experimental Committee of National Institutes of Natural Sciences (NINS; Nos. 08B005 and 10B001).

### Isolation of adult marmoset retinas

Animals were pretreated with medetomidine (0.01 mg/kg, i.m.; Domitol; Orion Corporation) and ketamine (10 mg/kg, i.m.; Ketalar; DAIICHI SANKYO ESPHA CO., LTD.) and were then overdosed with sodium pentobarbital intrapenetorially or intracardially (80–150 mg/kg; Somnopentyl; Kyoritsu Seiyaku Co, Ltd.). The eyes were removed and placed in carboxygenated (95% O_2_/5% CO_2_) Ames' medium (Sigma-Aldrich) prior to hemisection. The vitreous was removed with fine forceps. When it was difficult to remove the vitreous, opened eyecups were soaked for 10 min in hyaluronidase (0.07 mg/ml, Type IV-S, Sigma-Aldrich) -containing carboxygenated Ames' medium. Retinas were teased off the sclera and the pigment epithelium with fine forceps.

### Organotypic tissue culture of adult marmoset retinas

**Figure 1:**
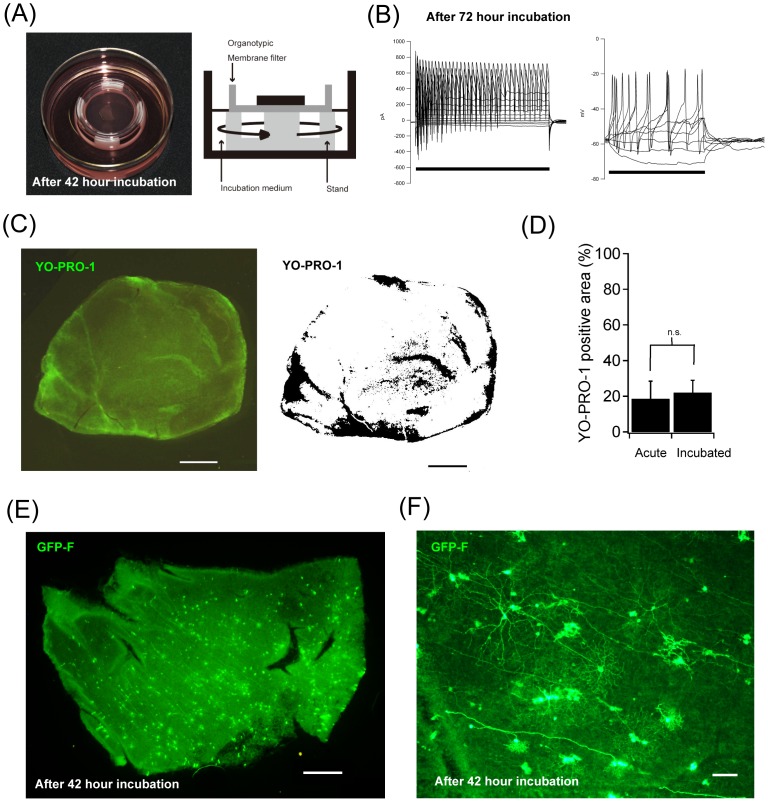
Incubation of adult marmoset monkey retina with transient gene transfection. (A) Organotypic culture of adult marmoset retina after 42-hour incubation. Left, a photograph of the interphase chamber of our organotypic culture system. Right, a schematic diagram of the interphase chamber. Deep dishes were used with custom-made stands to support the tissue culture insert with a flat-mounted retina. The retina was in contact with the medium over the filter on the photoreceptor side, and the ganglion cell side faced the atmosphere. Dishes were agitated by a rotary shaker in the CO_2_ incubator during the incubation. (B) Patch-clamp recordings of a cultured retinal ganglion cell after 72-hour incubation. Left: Voltage-clamp recordings. Holding voltage  = −71 mV. Voltages were clamped from −91 mV to +19 mV, in 10-mV steps, for 100 msec (bar). Right: Current-clamp experiment. Injected currents were from −20 pA to 50 pA, in 10-pA steps, for 100 msec (bar). Resting membrane potential was −59 mV. (C) A piece of marmoset retina after 3-day culture was stained with YO-PRO-1. YO-PRO-1-positive cells were clustered on the retina piece. The clusters formed an area with bright green fluorescence so that we defined a clustered area as a YO-PRO-1-positive area (black areas in left panel). Scale bars, 1 mm. (D) Quantification of YO-PRO-1-positive areas on cultured marmoset retinas. In the cultured retinas, 18.6±9.8% (mean ± standard deviation, n = 4 retina pieces) of the whole retinal area was YO-PRO-1-positive, while 22.0±6.0% (n = 4) of the retinal area was also YO-PRO-1-positive even in the acutely isolated retina (no significant difference). (E) A piece of *GFP-F*-transfected retina after 42-hour incubation. In this piece of retina, there were more than 452 cells expressing GFP-F. Scale bar, 1 mm. (F) Another piece of *GFP-F*-transfected retina after 42-hour incubation. Several retinal ganglion cells with different morphologies expressed GFP-F. Scale bar, 100 μm.

Adult marmoset retinal culture was perfomed according to the published protocol for the interphase culture system of adult rodent retinas [23] and adult rabbit retinas [24]. Marmoset retinas were placed ganglion cell side up on a 0.4-μm Millicell tissue culture insert (Millipore), and gentle suction was applied to the tissue for attachment to the membrane. Filter stands (3 cm in diameter, 1.2 cm in height) were cut from Delrin tubing so that the Millicell filter rested on stands when it was placed into a 60-mm diameter x 20-mm depth cell culture dish (“deep dish”; Nunc) (Figure 1A). More than 26 mL Ames' medium (Sigma–Aldrich) per retina (containing 0.192% sodium bicarbonate, 100 U/mL penicillin, 100 μg/mL streptomycin, and 0.292 mg/mL L-glutamine [Gibco]) was supplemented with 10% horse serum (Sigma–Aldrich), and then the medium was added to the dish. The retina was in contact with the medium via the Millicell filter on the photoreceptor side and with the incubator atmosphere (5% CO_2_, 37°C, humidified) over the ganglion cell side. During culture in the CO_2_ incubator, the medium was agitated constantly at 55 rpm using an orbital shaker (MIR-S100C; SANYO) and was exchanged daily.

### Labeling of ganglion cells by particle-mediated acute gene transfer of GFP-F

Gene gunning was carried out as previously described [23]. A Helios gene gun system (Bio-Rad) was used for particle-mediated acute gene transfer to retinal neurons. Ten mg Gold microcarriers (1.6 μm; Bio-Rad) was coated with 10 μg of CMV-EGFP-F plasmid [25] (gift from Dr. Noriyuki Kinoshita) in 3.2 ml ethanol solution and loaded into Tefzol tubing (Bio-Rad) using Tubing Prep Station (Bio-Rad) as described in the manufacturer's protocol. The gene gun barrel was held 5 mm above the retina and bullets were propelled at a delivery pressure of 110 psi.

### Cell viability assay for cultured retinas

To evaluate retinal cell viability after culture, we labeled cells undergoing apoptosis (and dead cells) with YO-PRO-1 (Invitrogen) after 3-day culture of marmoset retinas as previously described [23]. Images were captured using a fluorescence stereo microscope (SZX16; Olympus). Cells undergoing apoptosis and dead cells showed green fluorescence, and live cells showed little or no fluorescence (Figure 1C).

### Immunohistochemistry

**Figure 2:**
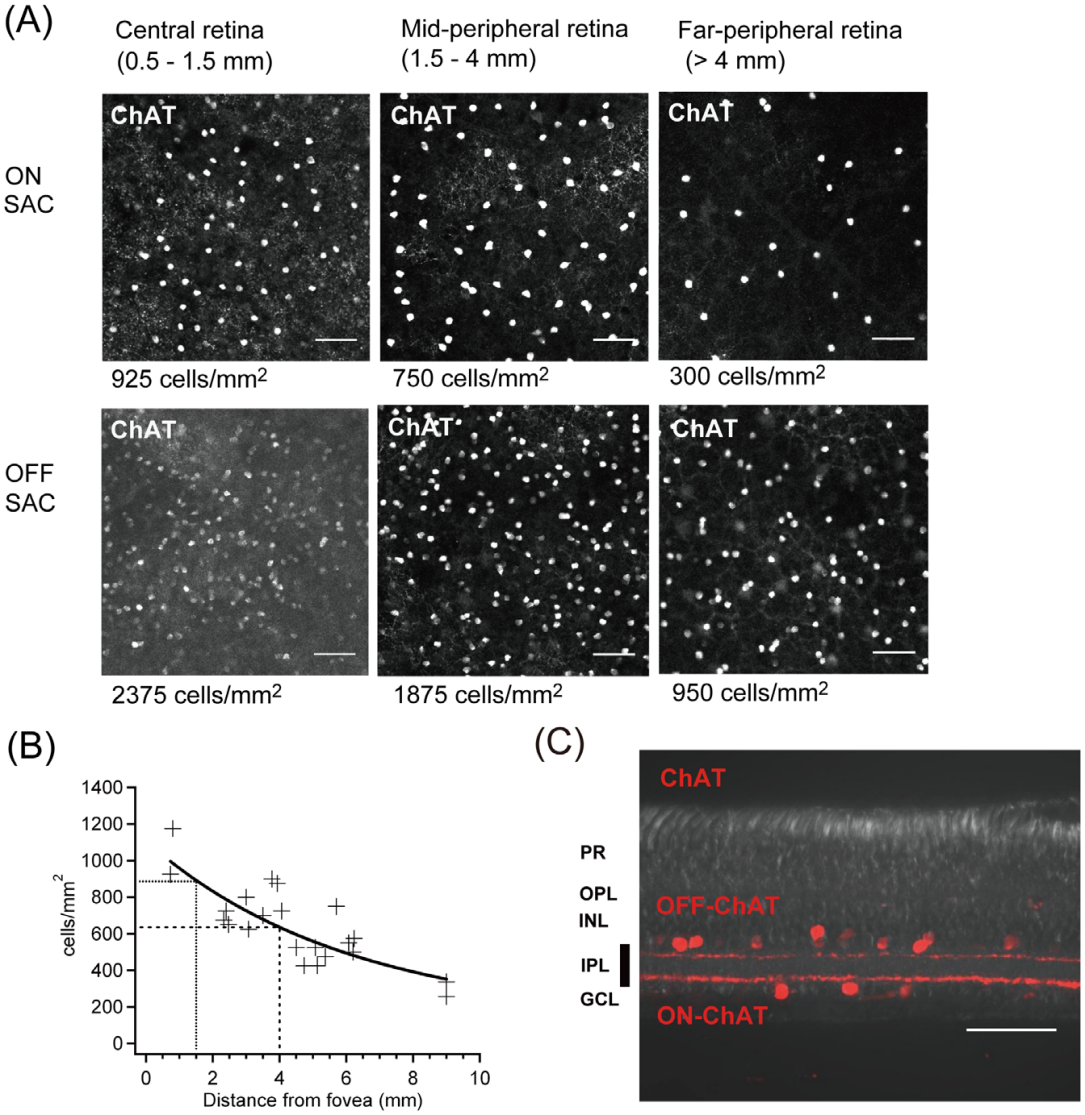
Eccentricity and stratification of ChAT-positive starburst amacrine cells. (A) ON and OFF ChAT-positive SAC in the central retina, mid-peripheral retina and far-peripheral retina. Scale bars, 50 μm. (B) Retinal eccentricity of ON SAC. From this graph, we defined the central retina (up to 1.5 mm), the mid-peripheral retina (>1.5 to 4 mm), and the far-peripheral retina (>4 mm). Scale bars, 50 μm. (C) Typical distinct stratifications of dendrites of ON and OFF ChAT-positive SAC in the IPL. These two bands are used as a landmark for checking stratification in the IPL. Scale bars, 50 μm.

Forty-two-hour-cultured retinas were fixed in phosphate buffered saline (PBS), pH 7.4, containing 4% paraformaldehyde for 1 hour at room temperature. For vertical sections shown in Figure 2C, the retinas were embedded in 4% agarose and 50-μm-thick sections were cut on a vibratome (Linearslicer PRO 10; Dosaka EM). The retinal preparations were rinsed with PBS and incubated for 1 hour at room temperature (sections) or at 4°C (whole mounts) in PBS with 4% donkey serum (Biowest) and 0.3% Triton X-100 (Katayama Chemical) for blocking prior to the addition of primary antibodies. Primary antibodies against ChAT (1∶200, goat polyclonal; Millipore) and GFP (1∶1000, rabbit polyclonal; Invitrogen) were used. The preparations were incubated in primary antibody solution overnight at room temperature (sections) or for 5 days at 4°C (whole mounts). After several rinses in PBS, preparations were incubated in secondary antibodies (donkey anti-rabbit IgG coupled to Alexa Fluor 488 and donkey anti-goat IgG coupled to Alexa Fluor 555, Invitrogen) diluted 1∶1000 in blocking buffer for 2 hours at room temperature (sections) or overnight at 4°C (whole mounts), rinsed, and coverslipped with Fluoromount-G or Dapi-Fluoromount-G (SouthernBiotech).

### Image analysis

Images were captured using a confocal laser scanning microscope (A1R; Nikon). Nikon PlanApo VC20x and 40x lenses were used. Stacks of confocal digital images at 0.2- or 0.5-μm z-axis spacing were acquired with appropriate filter sets. The brightness and contrast of the images were adjusted by using ImageJ (National Institute of Health) or Photoshop CS (Adobe). Confocal z-stacks were imported into Imaris software (Bitplane) and maximum intensity projection was performed using the section view.

Tracings of the dendritic tree and the soma of each ganglion cell were made using Neurolucida software (MicroBrightField) as previously described [26], [27].

### Patch-clamp recordings

Conventional recording techniques were used for patch-clamp recordings as described previously [23]. Briefly, patch pipettes (resistance of approximately 10 MΩ) were pulled from Pyrex tubing on a micropipette puller (P-97; Sutter Instrument). For whole-cell patch-clamp recordings, the pipette solution consisted of 125 mM K-gluconate, 5 mM KCl, 10 mM Hepes, 1 mM CaCl_2_, 1 mM MgCl_2_, and 11 mM EGTA (pH adjusted to 7.2 with KOH). A piece of retina was placed in a recording chamber, ganglion cell layer up, and continuously perfused at a rate of 1 to 2 mL/min with oxygenated extracellular solution (containing 125 mM NaCl, 2.5 mM KCl, 2 mM CaCl_2_, 1 mM MgCl_2_, 26 mM NaHCO_3_, 1.25 mM KH_2_PO_4_, and 12 mM glucose). The extracellular solution was continuously oxygenated with 5% CO_2_/95% O_2_ and kept between 32 and 35°C. The recording pipette was connected to the input stage of a patch-clamp amplifier, Axopatch 200B (Axon Instruments), and signals were sampled at 10 kHz with DigiData 1322A interface-type and pCLAMP8 software (Axon Instruments). The liquid junction potential was measured as 11 mV (Vm  =  Vp−11 mV) and corrected after recordings. Subsequent analysis was done by custom-made procedures in Igor Pro (WaveMetrics).

### Analysis of retinal eccentricity and stratification of dendrites by ChAT-positive starburst amacrine cells (SAC)

To determine retinal eccentricities and dendritic stratification in the inner plexiform layer (IPL) of *GFP*-transfected ganglion cells, each piece of the cultured marmoset retinas was stained with ChAT antibody, and density of ON SAC in each part was analyzed.

As in previous reports [21], [22], we use the term “foveal retina” to mean the part of the retina that deals with the first degree (radius) of visual angle from the fovea. The term “central retina” refers to the first 12° of visual angle (up to 1.5 mm in the marmoset), “mid-peripheral retina” refers to eccentricities of 12–31° of visual angle (>1.5 mm to 4 mm in the marmoset), and “far-peripheral retina” refers to eccentricities above 31° of visual angle (>4 mm in the marmoset).

Since not all of the small pieces of cultured retina included the fovea, retinal eccentricity of the observed cells was estimated retrospectively by determining the density of ChAT-immunopositive SAC in comparison with the density data for ChAT-immunopositive SAC as shown in Figure 2A and B. To obtain density data for ChAT-immunopositive SAC, one whole isolated retina including the fovea was immunostained by ChAT antibody (Figure 2A), and the densities of SAC in 22 different spots in the retina were measured and plotted in a graph (Figure 2B). From this graph, the retinal eccentricities of individual ganglion cells in each piece of cultured retina were retrospectively estimated. Then we defined the terms as follows: “central retina” refers to density of ON SAC of more than 900 cells/mm^2^ (up to 1.5 mm from the fovea by our estimation), “mid-peripheral retina” refers to density of ON SAC of 650–900 cells/mm^2^ (>1.5 mm to 4 mm by our estimation), and “far peripheral retina” refers to density of ON SAC below 650 cells/mm^2^ (>4 mm by our estimation).

**Figure 3:**
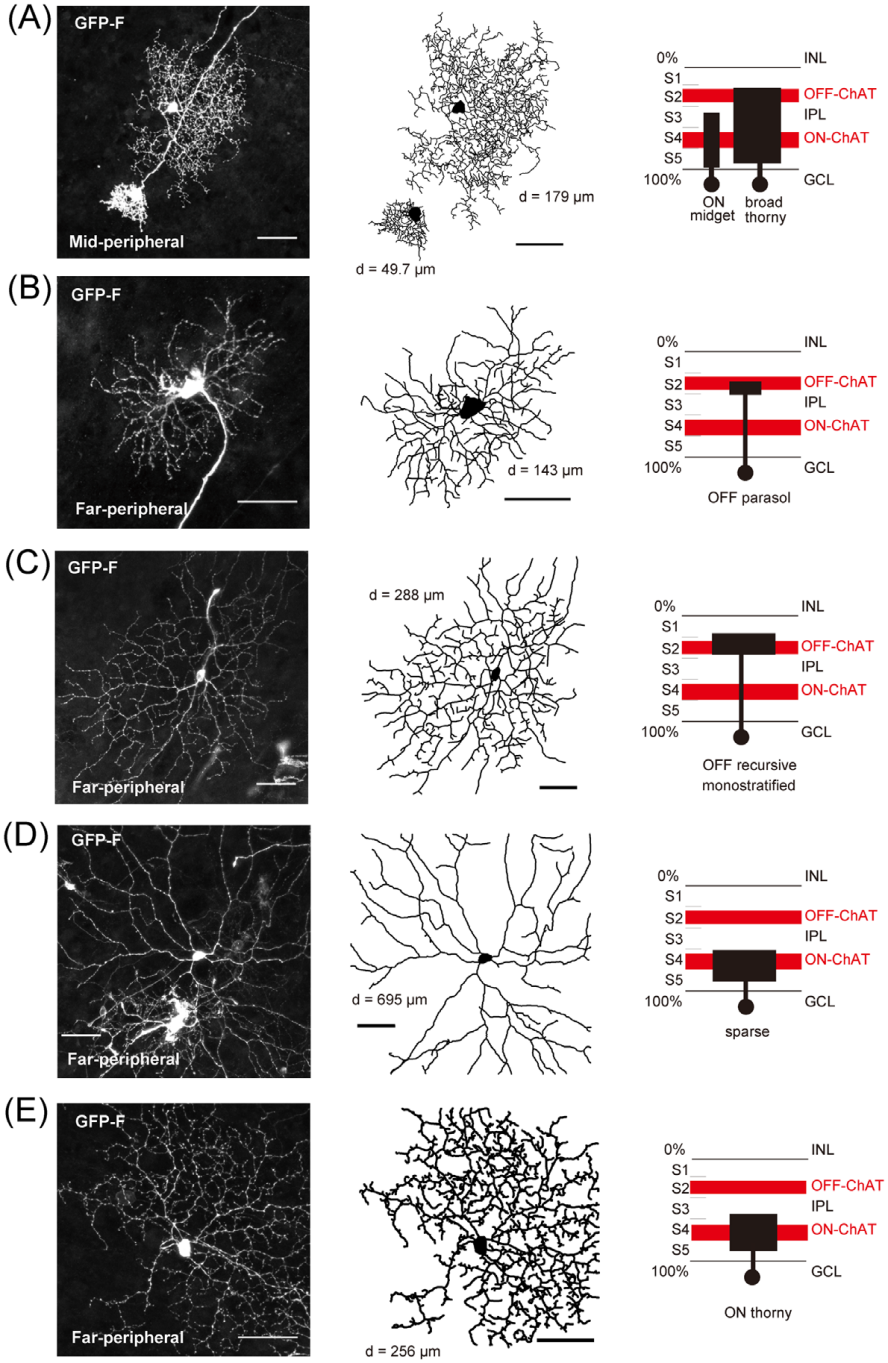
Morphological diversity of *GFP-F*-transfected monostratified or broadly stratified retinal ganglion cells. (A) An ON midget cell and a broad thorny cell (dendritic field size: 49.7 μm in diameter and 179 μm in diameter, respectively). (B) An OFF parasol ganglion cell (143 μm in diameter). (C) A OFF recursive monostratified ganglion cell (288 μm in diameter). (D) A sparse ganglion cell (695 μm in diameter). (E) An ON thony ganglion cell (256 μm in diameter). Left panels: projections of a confocal image. Middle: Tracings of dendritic structure. Right: Stratification of these ganglion cells compared with ON and OFF ChAT bands in the IPL. Scale bars, 50 μm.

**Figure 4:**
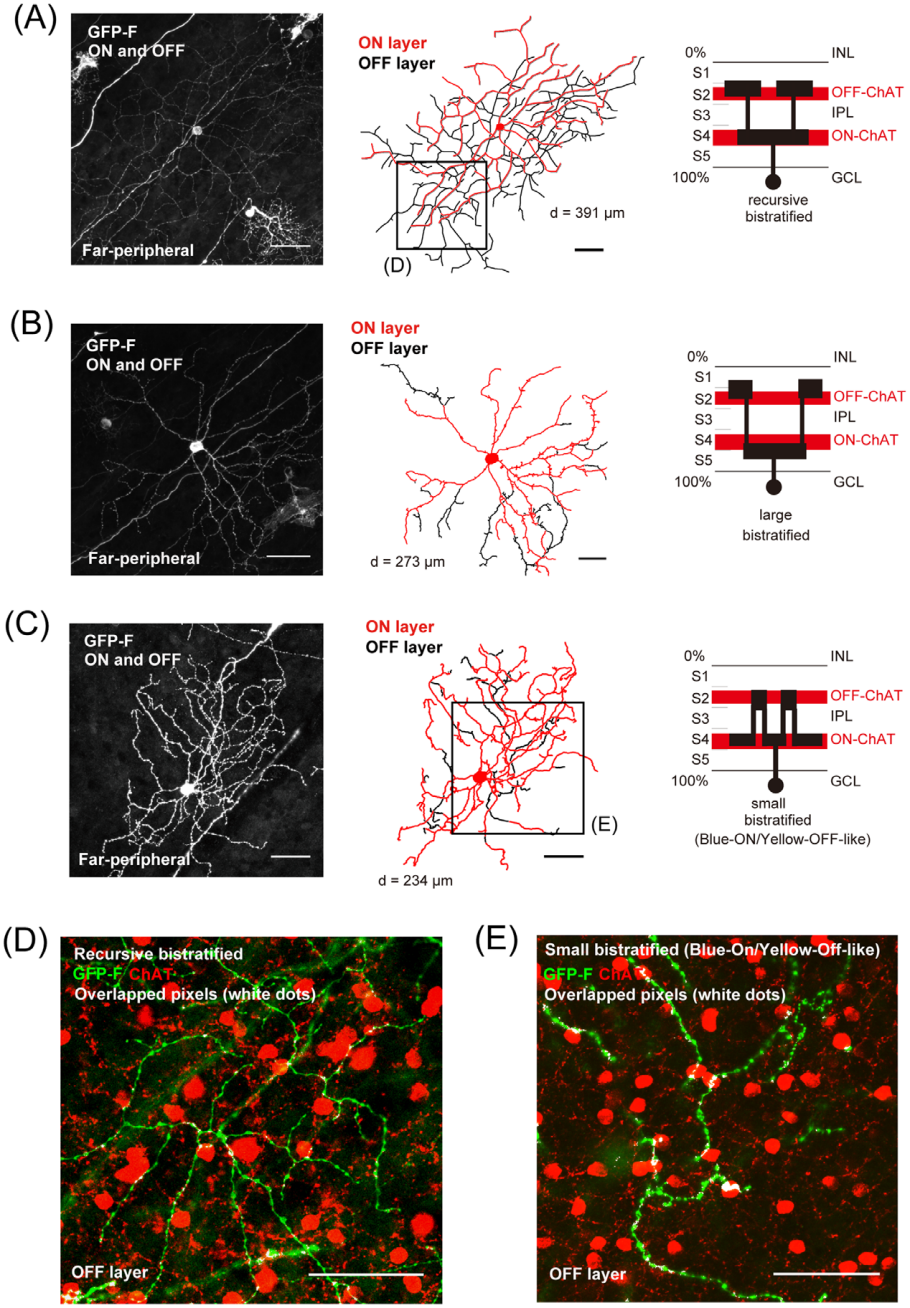
Morphological diversity of *GFP-F*-transfected bistratified retinal ganglion cells. (A) A recursive bistratified ganglion cell (dendritic field size: 391 μm in diameter). (B) A large bistratified ganglion cell (273 μm in diameter). (C) A small bistratified ganglion cell (Blue-ON/Yellow-OFF-like) (234 μm in diameter). Left panels: projections of a confocal image. Middle: Tracings of dendritic structure with ON (red trace) and OFF layers (black trace). Right: Stratification of these ganglion cells compared with ON and OFF ChAT bands in the IPL. (D) and (E) Highly magnified images of the OFF layer of the recursive bistratified ganglion cell (D; boxed area in A) and OFF layer of the small bistratified ganglion cell (E; boxed area in C) are shown. GFP-F-positive dendrites (green) were not obviously cofasciculated with ChAT-positive dendrites (red). Overlapped pixels are shown in white. The cofasciculation indexes (calculated on the OFF dendrites in D and E, see Materials and Methods) were calculated to be 0.55 and 1.08, respectively. Scale bars, 50 μm.

**Figure 5:**
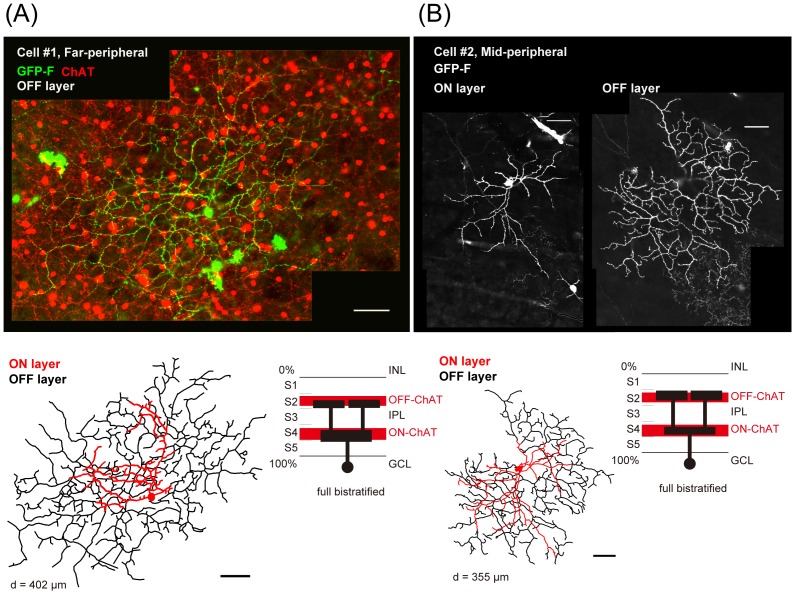
A candidate for bistratified direction-selective retinal ganglion cells in the marmoset monkey retina. (A) and (B) A *GFP-F*-transfected candidate for bistratified direction-selective retinal ganglion cells, namely Cell #1 (A), and Cell #2 (B) (dendritic field size: 402 μm in diameter and 355 μm in diameter, respectively). Confocal images, tracings and stratifications are shown. Scale bars, 50 μm.

In the vertical section of the retina (Figure 2C), ON and OFF SAC had typical distinct ON and OFF ChAT-positive bands in the IPL. These should be a landmark for analyzing the stratifying layer of dendrites in the IPL. Dendritic stratification in the IPL was defined as 0–100% from the border of the inner nuclear layer to the border of the ganglion cell layer and was measured at several locations in the dendritic tree in the confocal z-stack image. We used ON and OFF ChAT bands in the IPL as landmarks for identifying stratification (Figure 2C). A schematic drawing of dendritic stratification of each cell shown in Figures 3, 4, and 5 simply summarizes ramification depth (peak dendritic location) and thickness (dendritic width) in the IPL compared with ON and OFF ChAT bands.

To quantify dendritic field size of each ganglion cell, a convex polygon was drawn by linking the outermost tips of dendrites, and the area was measured by ImageJ (NIH). Then the area was converted to diameter by assuming a circular dendritic field (Figures 3, 4 and 5).

### Analysis of cofasciculation

**Figure 6:**
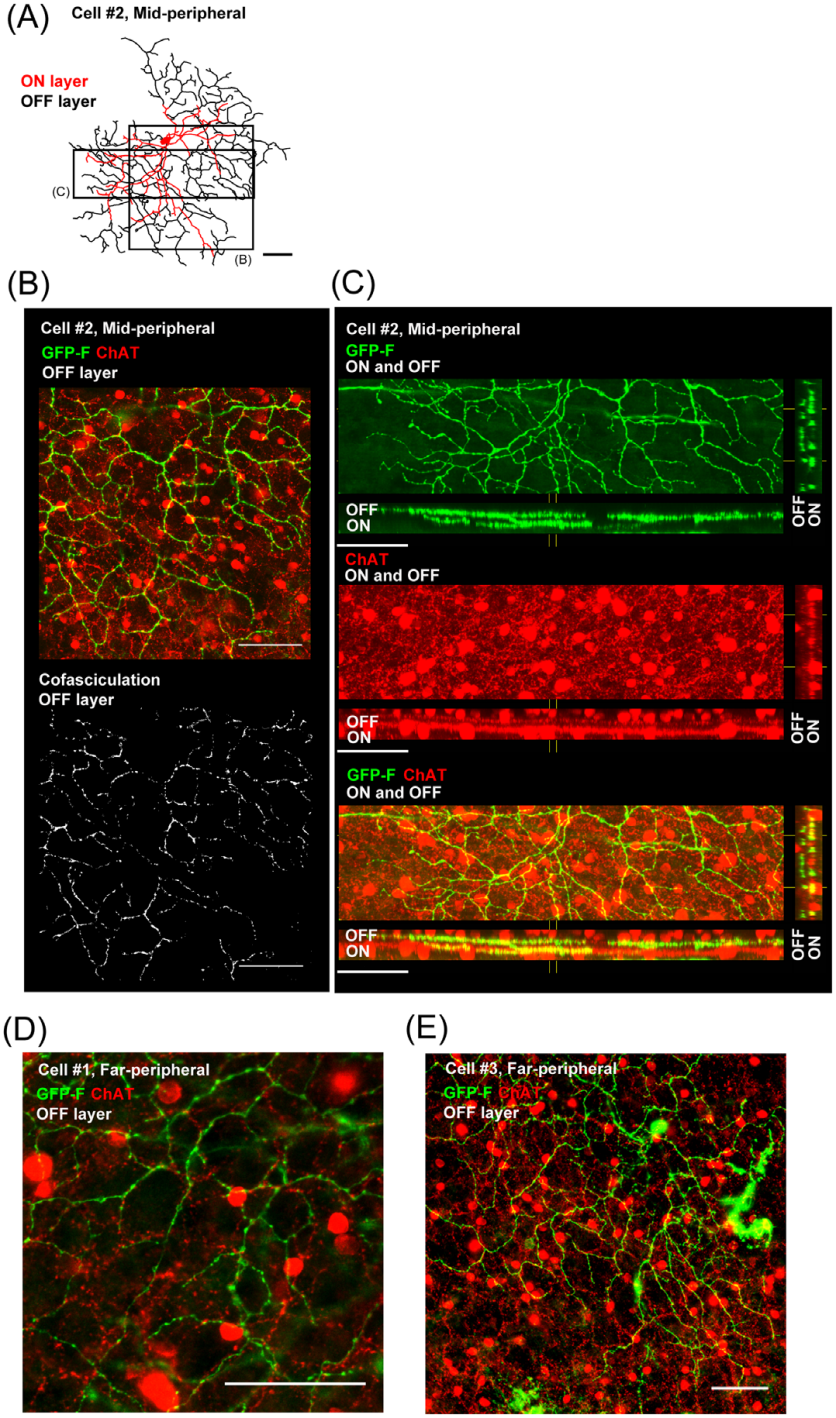
Cofasciculation and costratification of dendrites of the candidate for DSGC with ChAT-positive starburst dendrites. (A) A tracing of Cell #2 in Figure 5 with two boxes that represent the analyzed areas in (B) and (C). (B) GFP-F-positive dendrites (green) were cofasciculated with ChAT-positive dendrites (red) to form a honeycomb-shaped meshwork. OFF layer is shown here. Bottom panel: Only cofasciculated pixels are shown (analyzed by ImageJ). (C) GFP-F-positive dendrites (green) were clearly costratified with ON and OFF starburst ChAT bands (red) in IPL. (D) and (E) Highly magnified images of Cell #1 (D) and Cell #3 (E) are shown. GFP-F-positive dendrites (green) were clearly cofasciculated with ChAT-positive dendrites (red) to form a typical honeycomb-shaped meshwork. Scale bars, 50 μm.

To check the cofasciculation of dendrites between *GFP-F*-transfected cells and ChAT-positive starburst amacrine cells, shown in Figure 6, we calculated a cofasciculation index as previously reported [28]. Cofasciculation index (CI) was defined as follows:
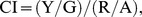
where A is the total number of pixels in the area analyzed, R is the number of pixels in the red channel (ChAT-immunopositive pixels), Y is the number of overlapping pixels (yellow pixels), and G is the number of pixels in the green channel (GFP-F-positive pixels). If the distribution of both channels is random, the probability of red pixels being in the whole analyzed area should be the same as that of red-and-green overlapping pixels in green-positive pixels (number should be 1). A larger percentage of overlapping pixels signifies a tendency towards cofasciculation.

All analyses were performed by ImageJ (NIH).

## Results

### Organotypic tissue culture of adult mamorset monkey retina with particle-mediated gene transfer

We successfully applied our organotypic tissue culture method [23], [24] with modifications for the adult marmoset monkey retina with transient gene transfection by particle-mediated gene transfer (Figure 1A, see also Materials and Methods). Briefly, we placed adult marmoset monkey retina tissue in our interphase culture system. In the interphase chamber, the retina was in contact with the medium over the filter on the photoreceptor side, and the ganglion cell side faced the atmosphere. During the incubation, the medium was agitated at 55 rpm by a rotary shaker in a CO_2_ incubator at 37°C. Particle-mediated acute gene transfer (“gene gun”) was conducted from the retinal ganglion cell side. In the interphase chamber, we successfully incubated adult marmoset monkey retina tissue for 2 to 4 days *ex vivo*. Although light responses were not observed after incubation for several days because of loss of photosensitivity under room light, retinal ganglion cells in incubated retina tissue on which patch clamping was successfully performed were all electrophysiologically active (n = 7) and showed action potentials responding to current injection as well as transient Na^+^ currents under voltage clamp recordings after 72-hour incubation (Figure 1B).

To check the cell viability of cultured retina, we labeled cells undergoing apoptosis (and dead cells) with YO-PRO-1 after 3-day (72 hours) culture of marmoset retina pieces [23], [24]. We found that YO-PRO-1-positive cells were clustered in cultured retinas and formed a distinct area with bright green fluorescence (Figure 1C). We defined a bright green area as a YO-PRO-1-positive area (Figure 1C) and quantified YO-PRO-1-positive areas on marmoset retinas in comparison with those on acutely isolated retinas. In the cultured retinas, 18.6±9.8% (mean ± standard deviation, n = 4 retina pieces) of the whole retinal area was YO-PRO-1-positive, while 22.0±6.0% (n = 4) of the retinal area was also YO-PRO-1-positive in the acutely isolated retinas (Figure 1D, no significant difference). More than 70% of the retinal area was still viable after 3-day incubation.

### Morphological diversity of *GFP*-transfected retinal ganglion cells

We transfected a *GFP* variant (*GFP-F*) [25] into organotypic tissue culture of the adult marmoset retina to survey the morphological diversity of RGCs. GFP-F is a variant of GFP that has membrane targeting signals to clearly label the membrane for showing detailed morphology of transfected cells.

As shown in Figure 2A and B, we first obtained density data for ON SAC in one isolated whole retina including the fovea and plotted the data in a graph (Figure 2B). From this graph, the retinal eccentricities of individual ganglion cells in each piece of cultured retina were retrospectively estimated. The density of ChAT-positive ON SAC was similar to that in human and macaque retinas [29]. Stratification of dendrites in the IPL was retrospectively determined by comparison with the depth of ChAT-immunopositive SAC as shown in Figure 2C.

We examined the morphology of 121 fully-labeled cells in the mid-peripheral and far-peripheral retina from more than 3240 *GFP*-transfected cells in 5 retinas (8.13 cell/mm^2^) as shown in Figures 1E and F. These 121 cells were selected because dendrites of these cells were not greatly overlapped with those of other GFP-positive cells. Ganglion cells were classified according to their morphology and stratification level in the IPL (see Materials and Methods). Among them, we identified various types of monostratified or broadly stratified ganglion cells shown in Figure 3 (midget, parasol, sparse, recursive, thorny, and broad thorny ganglion cells), and bistratified ganglion cells shown in Figure 4 (recursive, large, and small bistratified ganglion cells [blue-ON/yellow-OFF-like]) [7], [8]. Although the number of *GFP*-transfected cells did not imply actual density of the populations, we counted the cells of each subtype out of the 121 identified cells (Table 1). The midget cells were most abundant with 21 cells in the 121 identified cells (Figure 3A), and they were well-matched morphologically with those in a previous study on the marmoset retina [12], [14], [20], [30]. The parasol cells were second most abundant, 20 cells [12], [14] (Figure 3B). There were 13 broad thorny ganglion cells (Figure 3A), and they were morphologically similar to those in previous studies on the marmoset retina [12], [19], [20] and macaque retina [8], [31]. We identified 9 recursive monostratified ganglion cells [7], [8] (Figure 3C). We also identified sparse monostratified ganglion cells and thorny monostratified ganglion cells shown in Figure 3D and 3E, respectively, morphologically similar to those in previous studies on the marmoset retina [12], [16] and macaque retina [8], [32]. As bistratified ganglion cells shown in Figure 4 and Table 1, we found 10 recursive bistratified ganglion cells, morphologically similar to those in previous studies on macaque retina [33], and 5 large bistratified ganglion cells, morphologically similar to those in previous studies on the marmoset retina [16]. We also found 5 small bistratified ganglion cells (blue-ON/yellow-OFF-like) (Figure 4C), and they were morphologically similar to blue-ON/yellow-OFF retinal ganglion cells of the marmoset [13], [20], [34] and those of the macaque [31], [35]. Although stratifications of dendrites of recursive bistratified ganglion cells and small bistratified ganglion cells were close to ON and OFF ChAT-positive bands in the IPL, there was no obvious cofasiculation between GFP-positive dendrites of either type of bistratified ganglion cells and dendrites of ChAT-positive SAC as shown in Figure 4D and E.

**Table 1:** A list of 121 *GFP*-transfected marmoset retinal ganglion cells.

Monostratified or Broadly stratified (in Figure 3)	# of cells
Midget	21
Parasol	20
Sparse	14
Broad thorny	13
Thorny	10
Recursive	9
unclassified monostratified	7
Total	94

Bistratified (in Figure 4)	# of cells
Recursive	10
Large	5
Small bistratified (blue ON/Yellow Off-like)	5
A candidate for bistratified DSGC in this study (in Figures 5, and 6)	3
unclassified bistratified	4
Total	27

Taken together, the results obtained by using our organotypic tissue culture system show morphological diversity of *GFP*-transfected retinal ganglion cells in the marmoset monkey retina in addition to that of midget and parasol cells.

### A candidate for bistratified direction-selective ganglion cells

The next purpose of our study was to find a candidate for bistratified direction-selective ganglion cells (DSGC) [36] in the adult marmoset monkey retina. Before starting the search for bistratified DSGC in the marmoset monkey retina, we performed immunostaining to check for ChAT-positive SAC, which should interact with bistratified DSGC [28], [36]. As shown in Figure 2, ChAT-positive SAC were clearly stained in the inner nuclear layer (INL) and ganglion cell layer (GCL). Density of OFF SAC was much greater than that of ON SAC. ON SAC showed a decrease in density with retinal eccentricity (Figure 2B). Most importantly, two distinct ChAT-immunopositive bands were observed in the IPL, although OFF bands were weaker than ON bands (Figure 2C). Therefore, we used ChAT immunostaining as a reliable landmark to search for bistratified DSGC.

To find a candidate for bistratified DSGC, we searched for cells with the following criteria [28], [36]: (1) cells whose dendrites were stratified in the same layer as the dendrites of SAC, (2) cells whose dendrites cofasciculated with ChAT-immunopositive dendrites in both the ON and OFF sublaminae, and (3) cells with a dendritic arbor forming a characteristic honeycomb shape morphologically similar to that of rabbit bistratified DSGC [37]. Some researchers have tried to morphologically identify direction-selective ganglion cells in macaque monkey retinas, but cells that fully satisfied the above criteria have not been identified yet [32]. In the present surveying study, we found 3 cells of the 121 morphologically identified cells that matched these criteria. The cells shown in Figures 5 and 6 are candidates for bistratified DSGC. The dendrites in the ON and OFF layers of the IPL were costratified with the two distinct ChAT bands (Figure 5). Especially in the OFF layer, the dendrites were well cofasciculated with those of ChAT-positive starburst amacrine cells (Figure 6B). To statistically analyze cofasciculation of GFP-expressing dendrites and ChAT immunostaining shown in Figure 6B, cofasiculation index (CI) was calculated to be 1.43 (calculated on the OFF layer dendrites shown in Figure 6B, also shown in Figure 6A, Rectangle B, see Materials and Methods), where CI is the ratio of the probability of overlapping pixels on GFP-positive pixels to that of ChAT-positive pixels on all pixels of the analyzed area [28]. In Figure 6B (bottom panel), cofasciculated pixels on the dendrites are shown. In addition, the dendritic arbor seemed to form a honeycomb-shaped meshwork (Figures 5A and B, see also Figure 6D and E). Taken together, we concluded that these 3 cells as a subtype of bistratified retinal ganglion cells in the marmoset monkey retina are morphologically a strong candidate for bistratified DSGC.

## Discussion

Our organotypic tissue culture of the adult marmoset monkey retina with genetic engineering methods opens a new era of retinal neuroscience of the adult monkey retina. By combining with transient gene transfection using particle-mediated gene transfer, durable use of such a valuable material could be maximized. In the present study, an extensive survey of more than three thousands of retinal ganglion cells by *GFP* transfection revealed that diverse subtypes of retinal ganglion cells exist in addition to the dominant midget and parasol cells in the marmoset monkey retina. By this surveying method, we found a candidate for bistratified DSGC in the marmoset monkey retina. The advantage of the incubation and *GFP-F* gene transfection method is that hundreds of retinal ganglion cells per a retina piece can be labeled simultaneously. The method could be easily combined with immunohistochemistry. Thus, it enabled us to find a rare subtype of ganglion cells in the marmoset monkey retina as a candidate for directional selective bistratified ganglion cells whose dendrites were costratified and cofasciculated with ChAT-immunopositive bands. Since we used a CMV promoter for expressing GFP-F in this study, the gene expression itself was not selectively targeted for certain cell types.

Candidates for directional selective ganglion cells in primates have been previously suggested by their characteristic morphologies, such as “G11 cells” in the human retina by Kolb and coworkers [38]. As far as we know, this is the first report of bistratified RGCs with dendrites costratified and cofasciculated with ChAT dendrites in the marmoset monkey retina. By these characteristic features, we concluded that this type of bistratified cell is a strong candidate for bistratified directional selective ganglion cells. Meanwhile, we noticed that the OFF layer dendrites of these candidates shown in Figure 5 and Figure 6 were spread more widely and showed more obvious honeycomb shape than did the ON layer dendrites. In terms of ChAT bands, as shown in Figure 2C, ON layer ChAT bands were more strongly stained against ChAT than were OFF layer ChAT bands. These relatively large asymmetries in both the ganglion cells and ChAT-positive starburst bands differ from those in rabbits. Since there are in fact very large inter-species variations in types of retinal ganglion cells, we can't exclude the possibility that this cell could be another type of bistratified cell.

Electrophysiological recordings must be done to confirm this cell's functional property in the marmoset monkey retina. To do so, we need to overcome problems such as how to maintain light-responsiveness during the organotypic culture. Light-responsiveness tended to be diminished during the organotypic culture because the pigment epithelium was removed. One possible attempt could be to add retinal to the incubation medium to keep photocycles of photoreceptors active. If light-responsiveness can be successfully maintained, our genetic engineering method would provide a new approach to future investigation for morphological and also functional diversity of RGCs in the monkey retina.
